# How Does Professional Habitus Impact Nursing Autonomy? A Hermeneutic Qualitative Study Using Bourdieu’s Framework

**DOI:** 10.3390/nursrep15030088

**Published:** 2025-03-04

**Authors:** Laura Elvira Piedrahita Sandoval, Jorge Sotelo-Daza, Liliana Cristina Morales Viana, Cesar Ivan Aviles Gonzalez

**Affiliations:** 1 Department of Nursing, Universidad del Valle, Cali 700000, Colombia; laura.piedrahita@correounivalle.edu.co (L.E.P.S.); jorge.sotelo@correounivalle.edu.co (J.S.-D.); liliana.morales@correounivalle.edu.co (L.C.M.V.); 2 Department of Nursing, University of Studies of Enna Kore, 94100 Enna, Italy

**Keywords:** role of the nurse, nursing, nursing education, professional training, qualitative research

## Abstract

**Background/Objective:** In nursing practice, differences have been noted between the shared habitus acquired during academic training and professional practices within healthcare systems. In this context, nurses tend to experience an impact on their autonomy due to the ways in which their professional habitus has been established, which, in some way, alters the cultural capital acquired during their academic training. The objective of this study was to identify factors that facilitate and/or limit autonomy in nursing practice based on professional habitus. **Method:** This research was conducted using a hermeneutic qualitative study framed within a critical approach that incorporated Bourdieu’s theory of fields (habitus, field, and capital). This study included 11 registered nurses working in hospital settings, 17 nursing students, and six university professors. Data collection included 34 sociodemographic forms, 34 individual semi-structured interviews, and five *focus group discussions* conducted with an interview guide. The collected data were analyzed using an interpretative hermeneutic approach, integrating grounded theory and Bourdieu’s theory of fields, focusing on the concepts of habitus, field, and capital. **Results:** This study identified a central theme—clarification of the nurse’s role (professional habitus)—alongside three subthemes: (1) strengthening the nursing identity (identity habitus), (2) optimizing nursing education (optimization habitus), and (3) reinforcing professional credibility (validation habitus). Autonomy was found to be influenced by hierarchical structures, power relations, and institutional constraints within the healthcare social field, which led to limitations in the accumulation of nurses’ symbolic capital. **Conclusions:** The professional habitus of nurses is shaped by various elements within the healthcare social field. This field is constrained by hierarchical structures and factors such as subordination to the hegemonic biomedical discourse and the medical profession, limited recognition of humanized care, institutional restrictions on acknowledging the nursing process, and a lack of solidarity and leadership. These constraints ultimately hinder the accumulation of symbolic and social capital in nursing, leading to a loss of autonomy and hindering professional development.

## 1. Introduction

Registered nurses are considered the backbone of healthcare systems worldwide. Nurses’ persistence, commitment, and dedication are fundamental to achieving the core goal of providing safe, high-quality care to individuals, families, and communities [1]. Despite these considerations, tensions persist in nursing practice between the ideal discourse of care and its actual practice within the healthcare field [2]. These tensions arise from the discrepancies between the theoretical foundations acquired in academic settings and the realities of clinical practice, where registered nurses and licensed practical nurses may encounter situations that challenge their professional identity and autonomy.

In healthcare, notable disparities exist between the theoretical knowledge taught in nursing education and its application in practice. Disparities manifest through persistent stigmatization and undervaluation of the nursing role, excessive administrative workloads, limited participation in decision-making processes due to the hierarchical structure of healthcare systems, and the pressure to meet clinical performance indicators [3]. These factors significantly impact nurses’ professional performance, creating challenges and limitations in establishing comprehensive, high-quality care processes [2,3].

These structural conditions in healthcare make it difficult for nurses to apply their education fully, which ultimately restricts their independence and effectiveness in their roles [4].

The discrepancies between academic theoretical knowledge and professional practice (academic habitus vs. professional habitus) hinder the mobilization of cultural capital in nursing practice, thereby restricting professional autonomy and performance [2,5]. These constraints contribute to job dissatisfaction, difficulties in professional adaptation, and challenges to personal and professional identity. Furthermore, the limitations imposed by the healthcare field lead to a lack of professional recognition and restrictions on the nursing role. These conditions are often reinforced by dominant discourses and practices that prioritize economic and financial capital within healthcare systems, ultimately resulting in excessive workloads, reduced autonomy, and the reinforcement of subordinate positions for registered nurses and licensed practical nurses [6].

Exploring nurses’ experiences beyond the dominant biomedical discourse can provide a deeper understanding of the challenges related to their autonomy and reveal the interconnections between the factors shaping their practice. This calls for a more comprehensive analytical approach that considers the structural, cultural, and social dynamics influencing nursing practice.

The concept of habitus refers to internalized practices—that is, actions or behaviors that individuals perform automatically, having learned them through social dynamics. These practices define how individuals act, perceive, and think based on their lived experiences and social processes over time. Capital refers to resources that can generate power or advantages within a society; it is not limited to economic capital but also includes cultural capital (education, skills), social capital (relationships), and symbolic capital (status, prestige). The distribution of capital determines each individual’s position in society [6]. Field refers to the social arenas in which individuals and groups compete for various forms of capital, striving to attain positions of power that enable them to achieve their goals.

In nursing practice, the dynamics of habitus, capital, and field are interpreted through the lens of healthcare professionals, institutional structures, and power relations within the healthcare field. In this context, nurses have often been categorized as an oppressed group, dependent on the practices of other professions to structure their own care processes—ultimately limiting their autonomy [7]. Similarly, within the healthcare field, cultural capital is assigned based on institutional requirements, such as academic degrees, which determine the placement of professionals in prominent healthcare roles. Nurses often occupy a subordinate position within the healthcare hierarchy, which results in relationships characterized by limited authority, dependence, and reduced decision-making power [8].

This study aims to identify elements that may impact nursing professional practice by directly addressing the structural, cultural, and social factors that shape how nurses exercise their profession and make autonomous decisions within healthcare systems. Therefore, its potential contribution lies in providing insights to transform nursing professional practice, reinforce professional identity and roles, guide reforms in the healthcare and academic fields, contribute to structural changes in healthcare systems, and strengthen the nursing profession. The objective of this research was to identify factors that facilitate and/or limit autonomy in nursing practice based on professional habitus.

## 2. Methods

### 2.1. Study Design 

This study employed a qualitative, descriptive, and exploratory design. A critical and interpretative hermeneutic approach [9], based on Bourdieu’s theory of fields, was used. This approach enables an in-depth and critical analysis of individuals’ discourses and cultural practices, focusing on understanding their meanings [10]. A purposive sampling method was used to select participants [11].

Data collection was carried out using a methodological triangulation that integrated three instruments: (a) sociodemographic forms (n = 34), designed to characterize the social, economic, and demographic profiles of participants; (b) individual semi-structured interviews (n = 34), focused on exploring individual accounts and subjective experiences; and (c) focus group discussions (n = 5) with 31 participants in total, segmented into two sessions with nursing students (GF-NS1 and GF-NS2) that included 7 and 8 participants, respectively. Two sessions with clinical nurses (GF-CN1 and GF-CN2) were held, each with 5 participants. Finally, the group with university professors brought together 6 academics.

The sociodemographic forms operated as a contextualizing tool, linking individual trajectories with the phenomenon studied. Basic quantitative data were collected (age, gender, educational level, years of experience, institutional role, and socioeconomic context). This instrument was administered either by self-administering or with the support of the researchers during the initial phase of contact, guaranteeing confidentiality by assigning anonymous codes.

The individual semi-structured interviews (INT) prioritized interpretive depth through open questions and probing techniques. They were applied following a flexible thematic guide that combined predefined central questions. All participants were asked an initial question: “Can you tell me what aspects facilitate and/or limit autonomy in nursing practice?” Other questions included the following: “As a nurse, how do you perceive your autonomy in the context of your professional practice?”; “How does the institutional environment influence your perception of professional autonomy in nursing?”; and “Could you describe how you have dealt with these situations in your work?”. To encourage deeper responses, interviewers used probing comments, such as “Please continue”, “Tell me more about what this experience was like for you”, “What do you mean by this statement?”, “Can you think of anything else that might help me understand this experience?”, and “Is there anything else you would like to add?”. Key terms were explained in each interview to ensure conceptual clarity. The average duration was 45 min.

Focus group discussions (FGs) were organized with the purpose of identifying consensus, tensions, and nuances in collective perspectives, taking advantage of their interactive dynamics [12]. They were segmented according to the participants’ professional roles to ensure an environment that promoted free expression. Each session, lasting an average of 90 min, was moderated by a researcher trained in facilitation techniques, who stimulated discussion using trigger questions. The non-participation of some subjects in the focus group discussion was due to logistical constraints, such as incompatible schedules and prior commitments.

The combination of individual semi-structured interviews and focus group discussions responded to an intentional design to capture both the depth of individual subjective experiences and the social construction of meanings. Interviews were prioritized to access detailed personal accounts, exploring how participants internalize and negotiate their autonomy in specific contexts. Focus group discussions were used to reveal how norms, tensions, and hierarchies are collectively negotiated in nursing. Both techniques allow contrasting declared autonomy (in interviews) with performed autonomy (in group discussions), detecting contradictions between the professional ideal and actual practices.

The decision to integrate individual interviews and focus group discussions was based on theoretical–methodological pillars, aligned with the objectives and interpretations of the Bourdieusian framework [13], including the dual nature of the phenomenon studied, where autonomy in nursing is not only an individual act (linked to habitus and personal strategies) but also a collective phenomenon shaped by the rules of the professional field (institutional hierarchies, distribution of capital). While individual interviews captured how subjects internalize and resist these structures, focus group discussions exposed how the field regulates, normalizes, or questions these practices through group interactions. On the other hand, the integration of the two types of interviews leads to a triangulation to validate and deepen, where individual interviews offered hermeneutic depth (intimate meanings, unspoken contradictions), while focus group discussions provided dialectical richness (co-construction of norms, negotiation of power in real time).

The data obtained through the three collection methods were analyzed in a sequential and integrated manner, following a methodological triangulation approach [14]. Initially, each data set was processed separately to preserve the analytical specificity of each method. Subsequently, an analytical fusion was carried out using comparative matrices where the interview categories were crossed with the themes that emerged in the focus group discussions, identifying convergences, divergences, discursive gaps, and interpretive triangulation [14].

With the prior consent of the participants, all interviews and focus group discussions were audio-recorded and then transcribed into Word^®^ for the primary analysis of the data. The lead author conducted a pilot study of the interviews beforehand to ensure clarity and consistency in the sequence of the questions included in the data collection instruments.

This study was conducted at Universidad del Valle and Hospital Universitario del Valle in Cali, Colombia, from March to November 2021. Research participants included 17 nursing students (NS), 11 clinical nurses (CNs), and 6 university professors (UPs). Participants were selected based on their experience, professional roles within the institutions where the study was conducted, and their ability to provide unique perspectives on facilitators and barriers to autonomy. Eligible participants were required to be directly involved in nursing practice or education and have a minimum of two years of experience in clinical or academic settings. Researchers initially contacted potential participants via email, providing detailed information about the study objectives. Follow-up contact was made via telephone to provide further details, address any concerns, and coordinate interview times based on participant availability. Forty-two individuals were invited to participate in the study, of whom thirty-four agreed to participate and were recruited in a hospital setting.

### 2.2. Analytical Approach

This study was developed under a three-phase sequential methodological approach, integrating qualitative strategies to ensure a rigorous and multidimensional exploration of the analyzed phenomenon. In the first phase, Strauss and Corbin’s grounded theory [15] was implemented through a constant comparative analysis of empirical data collected through individual semi-structured interviews and focus group discussions to generate data-driven concepts. This process allowed the inductive generation of emerging conceptual categories articulated through open coding (in vivo identification of meaning units), axial (relationship of emerging categories), and selective (hierarchical theoretical integration) [15] means.

Subsequently, in the second phase, a hermeneutic interpretation (15) was adopted to deepen the understanding of the subjective meanings and discursive structures identified. Through an iterative dialogue between parts and whole (hermeneutic circle), the discourses were analyzed in their socio-historical context, prioritizing the understanding of inherent tensions and contradictions. Finally, in the third phase, Pierre Bourdieu’s (14) theoretical framework was integrated, using his notions of field, habitus, and capital to contextualize the findings within broader power structures and social dynamics.

Bourdieu’s field theory argues that society is organized into relatively autonomous fields (such as the health care system or academia), conceived as hierarchical social spaces in which agents compete to accumulate and distribute various types of capital (economic, cultural, social, and symbolic). Each field operates under a set of tacit rules and power relations that determine which practices, knowledge, and discourses are considered legitimate, while agents internalize these logics through habitus (historically acquired bodily and cognitive dispositions), unconsciously guiding their action strategies and enabling both the reproduction and transformation of the field’s structures. In the context of autonomy in nursing, this analytical framework allows us to understand how institutional hierarchies, struggles for professional recognition, and training paths (understood as forms of capital) shape autonomous practices, placing them in tension between individual agency (habitus) and the structural restrictions inherent to the health field, dominated by hegemonic actors (mainly physicians). Thus, the theory stands as a critical tool to denaturalize power relations and examine how autonomy is negotiated in a system of rules that are both objective and subjectively incorporated.

Each transcript of individual semi-structured interviews and focus group discussions was subjected to an iterative hermeneutic dialogue [16], where the parts (textual fragments) were contrasted with the whole (socio-institutional context), applying data matrices to systematize and classify the information.

The researchers coded the data jointly, identifying units of meaning (fragments of information that contain coherent and relevant ideas related to the phenomenon studied). These units of meaning were then grouped into broader categories based on emerging relationships.

The analysis involved multiple levels of interpretation: (a) Contextualization, which involved organizing and classifying information into data matrices (a methodological tool used to organize, systematize, and analyze the collected data). (b) Categorization, which consisted of theoretical coding (application of pre-established categories of the theoretical framework) to identify emerging categories through inductive coding during the analysis process. (c) Interpretive triangulation, which is related to the construction of content in line with the research objective. (d) Identification of emergent categories [12].

During the analytical process, the researchers held regular meetings to familiarize themselves with the data from the interviews and focus group discussions. The researchers, who were organized into cross-teams to avoid affinity bias, distributed the transcripts randomly, excluding analysis of their own interviews, and conducted iterative readings to identify categories. Discussions were held on key emerging themes and writing up the results. To represent the hermeneutic circle [17], a dialectical process was employed, iterating between emerging findings and individual interviews and conducting secondary readings of the original transcripts, which were evenly distributed among the researchers. Finally, the authors reached a consensus on the final primary and secondary categories.

Data from all three methods were managed in Atlas.ti 8.0, using hierarchical codes and semantic networks to visualize relationships between categories. Bourdieu’s theory [18] guided the final interpretation, analyzing how the nursing field (with its rules and hierarchies), professional habitus (internalized dispositions), and distribution of capital (symbolic, cultural) structure autonomous practices. Rigor was ensured through theoretical saturation, peer audits, and reflexivity in field diaries, documenting how the researchers’ positionality influenced the interpretation.

## 3. Results

The sociodemographic characteristics of the participants, including role, gender, age, educational level and professional experience, are detailed in Table 1.

**Figure 1:**
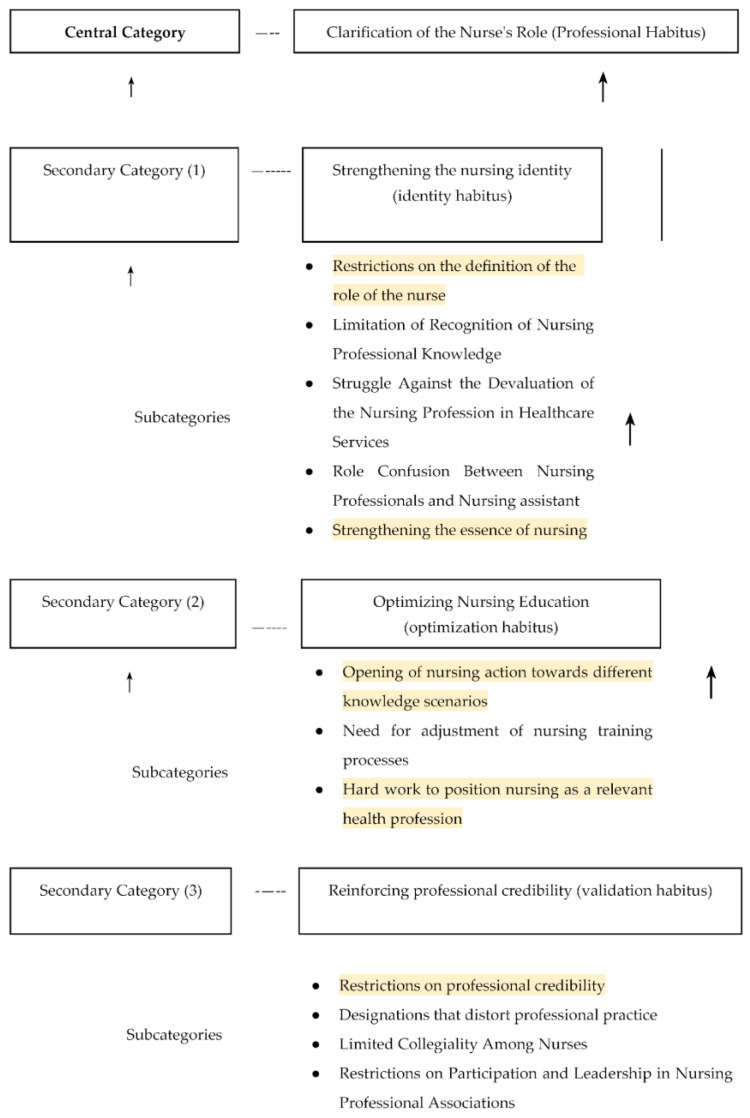
Categories and subcategories that facilitate or limit autonomy in nursing practice.

During the data analysis process, 275 codes emerged. A central category called clarification of the nurse’s role (professional habitus) was identified from this coding process (Figure 1). Similarly, three secondary categories emerged:

1. Strengthening the nursing identity (*identity habitus*);
2. Optimizing nursing education (*optimization habitus*);
3. Reinforcing professional credibility (*validation habitus*).

### 3.1. Central Category: Clarification of the Nurse’s Role (Professional Habitus)

Through the categorization process, the clarification of the nurse’s role emerged as the main thematic core. This category highlights the need for health policies and healthcare systems to explicitly define the roles, responsibilities, and functions of nurses across different professional settings. A clear and well-defined role is essential for strengthening and reinforcing nursing practice.

This issue is particularly relevant since the demands and restrictions imposed by healthcare systems may lead to limitations on nurses’ decision-making freedom in patient care processes, ultimately affecting their autonomy. Additionally, power relations and collaboration dynamics with other healthcare professionals and technicians may further constrain nurses’ autonomous practice.

Participant narratives revealed discourses, organizational policies, cultural practices, and structural barriers that may perpetuate limitations on professional independence and leadership.

### 3.2. Secondary Category: Strengthening the Nursing Identity (Identity Habitus)

#### 3.2.1. Restrictions in Defining the Nurse’s Role

The nurses who participated in this study described that a significant part of their professional practice revolves around following medical orders and performing administrative tasks. These practices are largely shaped by internalized dispositions that nurses develop through social and professional experiences (habitus), both during their academic training and in their clinical practice. According to participants, this habitus reflects the expectations imposed by the healthcare field, as well as the hierarchical structures that govern it. In this context, nurses’ cultural and social capital appears to be limited, as administrative efficiency and obedience tend to be prioritized over professional autonomy and critical thinking in nursing practice.

One participant emphasized this tension:

*“The tension is more about the meaning of the role we perform in clinics, in institutions [...] which society has come to believe is simply a nurse who follows orders, checks on patients, and is merely an administrative entity.”*

(FG-E1-006)

Another participant added the following:

*“The primary tension arises from the fact that, as an educator, I must instill critical thinking in students. That is, with the theoretical foundation they acquire, even if they have 70 patients, they must be capable of fulfilling their role as nurses, as part of a multidisciplinary team with a legitimate voice in decision-making. [...] The main cause of the tension between theory and practice is that we do not have a socially and politically well-defined role.”*

(INT-CN-002)

Based on the statements from professors and students, it is evident that the professional nursing role is poorly defined. This is reflected in varying interpretations of how nursing contributes to society. The lack of role definition not only affects how nurses perceive their own professional identity but also influences how society at large values and understands their importance.

#### 3.2.2. Limitation of Recognition of Nursing Professional Knowledge

According to the participants, nurses perform their professional work within healthcare systems by interacting with other healthcare professionals of the healthcare team, yet they are confronted by discourses of power that focus on the level of knowledge. In this context, the recognition of nursing expertise by other healthcare professionals remains limited.

One participant expressed this perception:

*“I believe that power is derived from knowledge [...] Medicine continues to disregard nursing knowledge [...] in that sense, they still see themselves as ’the masters’, and while we seek to break through, we have also maintained a submissive attitude that we have not been able to shed. They have their shield, and we have ours, but we have yet to establish real sources of recognition [...].”*

(FG-UP-004)

Additionally, participants frequently noted that nursing is continuously compared to medicine. According to them, this comparison is embedded within a hegemonic biomedical and patriarchal discourse, which devalues the care processes that define nursing practice. This hegemonic discourse affects both the perceived relevance of nursing care and the broader social perception of the nursing profession. One nurse articulated this issue as follows:

*“Nursing, as a field, has its own language, codes, body of knowledge, and methods of functioning that positively impact society. However, this is all part of the hegemonic discourse—nursing is always compared to medicine. In that comparison, nursing is devalued, because we are never compared in a way that highlights our strengths. The prevailing narrative is that physicians are the best. But we are different; we share a common element—human life—but each profession has its own domain [...]. The hegemonic discourse is patriarchal, it comes from a ’macho’ perspective, not even from a masculine one, but from a worldview where men dominate everything [...]. As a result, nursing care is undervalued because it is perceived as something inherent to women, as domestic work. This represents a major distortion of nursing’s image [...].”*

(INT-UP-002)

Participants’ narratives emphasize that dominant positions within healthcare institutions are primarily held by male specialist physicians, typically from upper-middle-class backgrounds and of mature age. These professionals establish a social hierarchy within healthcare settings, while women, younger individuals, lower-income professionals, and those with mestizo and Indigenous phenotypic characteristics tend to occupy lower stratified positions within the system.

#### 3.2.3. Struggle Against the Devaluation of the Nursing Profession in Healthcare Services

The participating nurses highlighted the need to combat the devaluation of the nursing profession within the healthcare field. They emphasized that this effort should originate from academic settings, where they have often perceived that even professors compare nursing to the field of medical knowledge, distorting the roles of both professions, which are inherently different.

One participant expressed this concern as follows:

*“Something that has bothered me since my first semester—and that happens frequently here, especially in the first five semesters—is that we are constantly being compared to medicine in every class. Here, they tell us things like: ‘We are better than medicine’, when in reality, we are different. We are a team; we need each other.”*

(INT-NS-007)

#### 3.2.4. Role Confusion Between Nursing Professionals and Nursing Assistants

Participant narratives indicate that nursing is often only partially recognized as a professional discipline. The nursing habitus, shaped by education and professional experiences, is in constant tension with the dominant habitus of other healthcare professionals. As a result, the role of professional nurses is frequently confused with that of nursing assistants.

One participant described this issue as follows:

*“Doctors and other healthcare professionals believe that nurses perform the same duties as nursing assistants. We constantly have to remind them that we graduated from a university and possess different competencies to care for patients based on professional healthcare actions.”*

(FG-UP-003)

Another participant emphasized the following:

*“The healthcare team is still unaware of everything a nurse can do across different professional fields—whether in hospitals, community care, teaching, research, or even policymaking.”*

(FG-UP-006)

Additionally, participants pointed out that the lack of recognition of nursing as a professional discipline results in a lack of clarity in distinguishing nurses’ roles, practices, and contributions to health processes. This devaluation of nursing expertise leads to symbolic and cultural capital being less valued compared to that of other healthcare professionals, creating an imbalance in the distribution of capital and reinforcing the invisibilization of nurses’ roles, competencies, and contributions.

One participant described this imbalance:

*“I feel that a major limitation is that physicians do not acknowledge the difference between a professional nurse and a nursing assistant. When they refer to ‘the nurse’, I always have to clarify: ‘Are you talking about the nursing assistant or the registered nurse on duty?’ And then they realize—‘Oh, I was referring to the assistant.’ So I remind them that their roles are different.”*

(INT-P-002)

Another participant added the following:

*“Another thing I’ve noticed is that when a nurse is confident and has strong clarity in their actions, it is perceived as ‘stepping on doctors’ toes’, so they don’t receive due recognition. I experience this firsthand because doctors always emphasize their own contributions, while what nurses do tends to be overlooked. In this department, I have managed to establish a strong leadership presence, and we have achieved effective teamwork. However, not all nurses have the opportunity to reach that level. When I integrate all these nursing care actions to achieve positive patient outcomes, both from a medical and nursing perspective, the collaboration is highly valued. We coordinate well, communicate effectively, and work together toward that goal.”*

(INT-CN-005)

#### 3.2.5. Strengthening the Essence of Nursing

Anchored in the nursing care process, participants recognized that strengthening professional development is intrinsically linked to the growth of nursing autonomy. This autonomy is sustained by the essential actions of comprehensive care provided to individuals in all their dimensions, moving away from dependence on the medical social field.

One participant expressed this perspective as follows:

*“Nursing is a field that must grow in parallel with the evolving nature of the human experience to address increasing complexities. We should not simply follow medical technology advancements [...]. In reality, nursing encompasses all domains concerning the human condition, which means we must keep pace with all forms of knowledge, whether from the social sciences or biological sciences.”*

(FG-CN2-003)

A notable concern described by participants was that, despite advancements in nursing disciplinary knowledge, some academic training programs continue to prioritize biomedical content while limiting courses based on humanistic and social epistemologies, as they are perceived as less relevant to healthcare education.

### 3.3. Secondary Category: Optimizing Nursing Education (Optimization Habitus)

#### 3.3.1. Opening of Nursing Action Towards Different Knowledge Scenarios

The participants’ stories reflect the need for nursing professionals to establish specific fields of work and, in this sense, manage to move away from the hegemonic discourse mobilized by medical specialties. One participant described it as follows:

*Beyond the medical specialty, I believe we have to think about the phenomena that concern us […] right now; for example, there is an important role in nursing care for coexistence for peace. What are we going to do about that? That generates new tools and the demand to investigate and think about ourselves in a social production of health. In Latin America, we have made many gains in understanding how our communities have developed in the inequitable relationships that have led to suffering and the conditions we have today. Nursing cannot continue to be alien to this knowledge.*

(GF-EA1-006)

#### 3.3.2. Need for Adjustment of Nursing Training Processes

Nurses describe the need to adjust nursing academic program settings to categorically establish the roles that characterize the discipline. One participant expressed it this way:

*It is up to us to find out what I am looking for when I provide care. I want to grow in providing better care, taking into account the patient’s needs at that moment, and I have more power when I can satisfy the needs of the overwhelmed person. That is why the humanization part is very well done.*

(GF-E1-009)

Another participant expressed the following:

*It is not that because of the doctor’s diagnoses, we have different ways of seeing our practice. He is made to diagnose, so what is the hegemony? I am made to care; if I am doing well what I am supposed to do, there is no problem there. It is my responsibility to care as a nurse, which is my discipline: caring. That should interest us and what we have to teach our students […] I believe that we have to create another form of education.*

(ENT-P-004)

#### 3.3.3. Hard Work to Position Nursing as a Relevant Health Profession

The participants’ stories show that nursing can achieve a high professional level like other disciplines, as long as effort and dedication are strengthened by strengthening the care process.

*I have had the opportunity to be in different roles; I have even been on the boards. I am the only nurse who has had that opportunity here in the institution, but much to my regret, that opportunity is increasingly being lost. I feel that this is part of our responsibility because if I had had recognition, it is also because many have perceived my work as a nurse, or else, I would not have gotten there […] In most cases, we assume roles of convenience, and there is no dedication.*

(ENT-EA-005)

### 3.4. Secondary Category: Reinforcing Professional Credibility (Validation Habitus)

#### 3.4.1. Restrictions on Professional Credibility

Nursing students participating in the research described that during their training process, some professors do not provide them with input that would lead them to strengthen the credibility of the profession. One student mentioned the following:

*We have teachers who do not value their profession, so, for example, I am writing my notes, and I want to make a nursing diagnosis related to the fact that the patient has an alteration in gas exchange. Hence, the teacher comes and tells someone:—That thing about gas exchange is from respiratory therapy, so don’t go there, take it away -, and that thing about arterial blood gases? Did you analyze them? Or did you see that the doctors did it, and you copied it?*

(GF-E2-002)

#### 3.4.2. Designations That Distort Professional Practice

According to the participants, one aspect that limits nurses in developing their professional practice is their labeling as “bosses”; this denomination leads to professionals being associated more with administrative functions than with direct care actions.

*I think it weighs heavily on us, and we must continue fighting against that view of being bosses because it is a view of the military that gives orders. Nursing leadership has to go beyond a power relationship because, often, having a staff member in charge as an auxiliary seems to be the status that we socially seek, which is different.*

(GF-EA1-010)

#### 3.4.3. Limited Collegiality Among Nurses

Participants point to the need for more collaboration between nursing professionals as a limitation in strengthening the discipline.

*It has to do with what we reflect from the training with the students; if a colleague does not do well with the students, how many of us are willing to ask her: what is happening? How do we support you? […] We are always in the sense of “competition”, of saying, you are good, you are bad, and then, if we ourselves, as in the teaching exercise, are not supportive of the other, we are impregnating that same thing in our students.*

(ENT-EA-008)

#### 3.4.4. Restrictions on Participation and Leadership in Nursing Professional Associations

Participants reported restrictions in strengthening nursing leadership, both in academia and in the workplace. In these settings, participation in nursing associations is weakened, limiting nursing action from associations that allow for political influence in improving the disciplinary foundations of the profession.

*I am still determining the role the ANEC (National Association of Nurses of Colombia) union plays. I think there should also be, I do not know, a redefinition, a collective construction, or something that leads us to think about and build a different union […]. If we lack it, perhaps some external help from these organizations can strengthen our leadership.*

(ENT-EA-008)

## 4. Discussion

In the process of identifying factors that facilitate and/or limit autonomy in nursing practice, various challenges and obstacles arise within the healthcare field, impacting nurses’ ability to effectively perform their professional roles, which are shaped by habitus, field, and capital.

### 4.1. Habitus and Autonomy in Nursing Practice

Nurses’ perception of their professional role and habitus within the healthcare field is influenced by both their academic training experience and professional trajectory. These aspects represent key resources in defining their ability to mobilize nursing’s cultural, symbolic, and social capital within the healthcare field. This study reveals limitations in nurses’ professional performance due to the lack of a clearly defined professional role, aligning with previous studies [7,19,20], which highlight how this ambiguity contributes to the devaluation of nurses’ symbolic and cultural capital [21].

Participants argue that nurses experience a subordinated professional identity, which ultimately restricts their autonomy in care processes. This finding is consistent with Moya et al. [8], who state that historically, nursing has been perceived as a discipline where professionals tend to adopt subordinate roles. This phenomenon is reinforced by the incorporation of biomedical, Euclidean, and positivist discourses into the nursing habitus, which favor disease-centered approaches (morbidocentric processes) while undermining the sovereignty of nursing knowledge from the perspective of humanized care.

Although nursing has progressively consolidated its disciplinary knowledge, it is important to note that this process has also led to the suppression of epistemological approaches from the human and social sciences in university curricula and research [22]. This phenomenon occurs, among other reasons, due to the need to meet the predominantly biomedical and economic demands of healthcare institutions, which operate under hegemonic discourses and practices focused on administrative and financial concerns while neglecting the human interaction required in nursing care [23]. These constraints may compromise nurses’ professional habitus, leading to weakened autonomy and limiting their ability to act within a humanized care framework.

Nurses’ professional habitus may be challenged by the dominant discourses and practices within the healthcare field, which can further impact their autonomy. However, nurses themselves play an active role in shaping their professional identity. This dynamic and relational nature of nursing practice is constructed through individual experiences, collective learning, ethical values, attitudes, and behaviors, which are essential components of their identity as caregivers. Additionally, interactions with social, cultural, and organizational processes enable nurses to reinterpret and redefine their role, shaping a professional habitus that aligns with the expectations of their specific context [24].

### 4.2. The Healthcare Field and Autonomy in Nursing Practice

The healthcare field in which nurses operate is shaped by power dynamics, where different actors and professions compete for specific forms of capital: symbolic (prestige), cultural (knowledge), social (networks and connections), and economic (financial resources). These power relations influence interactions among healthcare teams, shaping both opportunities and limitations in achieving nursing autonomy. Within this social field, challenges persist in establishing nursing as a recognized and influential profession, capable of systematically advancing to strengthen its cultural and symbolic capital and position itself as a highly valued discipline within society.

In this study, nurses perceived that within the healthcare field, there are ongoing criticisms from other professionals regarding the limited recognition of nursing as a profession, its scope, and its roles. These findings align with previous research [25], which highlights that nursing’s constrained professional recognition and low symbolic valuation contribute to a subordinated habitus. This subordination presents a challenge to the development of an autonomous nursing practice, hindering the discipline’s ability to consolidate symbolic capital within the healthcare field [26], which has historically been dominated by biomedical prestige. Furthermore, within this field, nursing’s role is not only undervalued but is also actively prevented from advancing due to the actions of other healthcare actors who hold greater power and symbolic capital [27].

One of the key factors influencing nurses’ position in the healthcare field is the feminization of the profession. Connell [28] supports this claim by describing how the work and practices carried out by women in healthcare settings are relegated to lower-power positions. This issue directly affects the nursing discipline, given that the majority of its workforce is female. Such gender-based discrimination fosters unequal power relations, where men, as the dominant group, perpetuate the subordination of women through discourses and practices that reinforce their perceived superiority. These unjust inequalities gradually become legitimized within the healthcare field, primarily as a result of the devaluation of the nursing care process.

Moreover, previous studies [29] have highlighted that nursing undergraduate students enter their programs with high expectations regarding their scientific training. However, their level of satisfaction declines due to the challenges they encounter in professional practice [30]. This decrease in satisfaction is, in part, explained by the lack of clarity surrounding the professional role of nursing care within the healthcare field, which ultimately restricts the accumulation of symbolic capital and prevents the consolidation of nursing as an authoritative presence within this social field.

### 4.3. Capital and Autonomy in Nursing Practice

The educational process in nursing plays a fundamental role in shaping nurses’ cultural and symbolic capital, as well as in defining their habitus. Research has shown that this setting often reproduces dependency practices, which ultimately limit the autonomy of the discipline [31]. This issue calls for critical attention regarding the educational projections for current nursing students, as it is essential to establish pedagogical structures that emancipate nursing from discourses that reinforce subordination and obedience.

Certain educational models, such as “Banking Education”, exemplify academic approaches that perpetuate dependency, limiting the development of a critically oriented cultural capital in nursing and restricting the possibility of transforming the healthcare field. This model focuses primarily on the transmission of knowledge, neglecting reflective processes that challenge the established power structures within healthcare hierarchies [32]. Moreover, such models fragment education, disregarding the social contexts in which healthcare realities unfold [33]. This fragmentation contributes to dissatisfaction in the educational process, as it disrupts the alignment between theory and practice.

When academic practice becomes fragmented [34], theoretical components are weakened, diminishing the recognition of the holistic nature of life settings, especially when viewed through a positivist habitus. This fragmentation imposes obstacles to contextualized analysis of health and care challenges, limiting the understanding of the deeper meanings underlying the accumulation of cultural capital in nursing. Furthermore, a strictly positivist habitus in patient care not only devalues core nursing principles but also reduces individuals to quantifiable entities, reinforcing economically driven priorities within the healthcare field. This economic emphasis significantly undermines the humanistic foundations that constitute nurses’ cultural and symbolic capital [35].

To transform the structural limitations imposed on nursing as a discipline, it is necessary to promote a critically reflective habitus in nursing education [36]. This habitual transformation should challenge the established power dynamics within healthcare systems in order to build symbolic and cultural capital that strengthens nursing’s recognition and autonomy [37]. Achieving this goal requires revisions to traditional educational models, ensuring a stronger focus on the essence of nursing care while resisting administrative and bureaucratic influences that have historically impacted the establishment of nurses’ cultural capital.

### 4.4. Proposed Solutions and Recommendations 

Based on the emergent categories identified in this study, several potential solutions can be proposed to address the challenges highlighted in the research. These include the following:

- Expanding academic training, strengthening nursing research, and disseminating the impact of the discipline;
- Adjusting the structure of nursing education programs to go beyond technical skill development, ensuring that students acquire critical thinking, ethical reasoning, and leadership abilities that enable them to recognize themselves as political agents with autonomy within the healthcare field;
- Integrating practical experiences that encourage reflection on the relevance of the nursing role, professional values, and contributions to patient care;
- Transforming workplace environments to ensure that nurses have the necessary conditions to practice autonomously and effectively, supported by health and organizational policies that respect and value their clinical judgment and decision-making, as well as fair compensation;
- Balancing administrative workloads and excessive working hours, allowing nurses to focus on delivering high-quality patient care.

### 4.5. Strengths

Among the strengths of this research, this study identified various categories through a critical approach that highlights factors limiting nursing autonomy. These categories provide valuable insights for analyzing nursing practice from labor, political, social, and cultural perspectives, contributing to the development of healthcare policies that support the nursing profession. Additionally, the findings offer guidance for future research and contribute to the identification of strategies to clarify and strengthen the nursing role, ultimately promoting nurse empowerment and the consolidation of professional autonomy in daily practice.

### 4.6. Limitations

A key limitation of this study is that it was conducted among a specific group of nurses and nursing students in Colombia, meaning that the narratives and experiences reported reflect a localized context. To achieve greater generalizability, future research should consider expanding the sample size and including diverse healthcare settings in the analysis.

## 5. Conclusions

This study reveals that nurses’ professional habitus is shaped by various elements within the social field of the healthcare system. This field is constrained by hierarchical structures that restrict nursing autonomy. Factors such as subordination to the hegemonic biomedical discourse and the medical profession, insufficient recognition of humanized care, limitations in the institutional acknowledgment of the nursing process, and a lack of solidarity and leadership within the profession can hinder the accumulation of symbolic and social capital in nursing. These limitations ultimately contribute to a loss of autonomy and barriers to professional development.

To mitigate the erosion of nursing autonomy, it is essential to strengthen a critical professional habitus, encouraging reflection from both the academic and healthcare social fields on the urgent need to reinforce the nursing role. 

Strengthening the nursing role requires encouraging the active participation of nurses in interdisciplinary committees, decision-making processes, and the design of institutional policies; enhancing the leadership of nursing associations; conducting research focused on real challenges of nursing practice; and developing leadership training programs for health system administrators that emphasize the integral comprehensive role of nurses and optimize digital processes to redistribute administrative tasks, allowing nurses to focus more on direct patient care.

A call to action is made to the nursing profession, emphasizing the need to strengthen the ongoing struggle against the devaluation of the discipline and reaffirm its core essence. This requires advocating for favorable work environments that uphold comprehensive patient care, recognize and respect nursing autonomy, value nursing-specific knowledge, and appreciate the cultural capital of the discipline.

## Figures and Tables

**Table 1:** Characteristics of the participants.

Role	Profession/Gender	Quantity	Age (Years)	Highest Level of Education Attained	Teaching Experience (Years)
**Professor**	Female Nurse	5	38–67	Master’s (5)	10–30
	Male Nurse	1	43	PhD (1)	22
**Clinical Nurse**	Female Nurse	10	30–61	Master’s (9); PhD (1)	3–27
	Male Nurse	1	38	Master’s (1)	16
**Nursing Students**	Female	11	21–24	High School (11)	None
	Male	6	21–24	High School (6)	None

## Data Availability

The data presented in this study are available from the corresponding author upon request.

## Public Involvement Statement

No public involvement in any aspect of this research.

## Guidelines and Standards Statement

This manuscript was written following the recommendations of the COREQ guide for qualitative research reporting (Tong et al., 2007 [38]).
